# Cannabinoid Modulation of Memory Consolidation in Rats: Beyond the Role of Cannabinoid Receptor Subtype 1

**DOI:** 10.3389/fphar.2017.00200

**Published:** 2017-04-12

**Authors:** Patrizia Ratano, Maura Palmery, Viviana Trezza, Patrizia Campolongo

**Affiliations:** ^1^Department of Physiology and Pharmacology, Sapienza University of RomeRome, Italy; ^2^Department of Science, Section of Biomedical Sciences and Technologies, Roma Tre UniversityRome, Italy

**Keywords:** endocannabinoid system, inhibitory avoidance, WIN55, 212-2, URB597, memory retention, emotional arousal

## Abstract

The effects induced by exogenous manipulation of endocannabinoid neurotransmission on emotion and memory are often contradictory. Among the different factors involved, of particular interest is the binding affinity of endocannabinoids, and their analogs, for other receptor families beyond cannabinoid receptors, such as the peroxisome proliferator-activated receptors (PPARs), and the transient receptor potential cation channel subfamily V member 1 (TRPV1). The aim of this study was to investigate which receptor subtype mediates cannabinoid effects on memory consolidation for emotionally arousing experiences. We tested two cannabinoid compounds with different pharmacological properties in the inhibitory avoidance task, and evaluated whether the observed effects are mediated by cannabinoid, PPARα or TRPV1 receptor activation. We found that the synthetic cannabinoid agonist WIN55,212-2 and the FAAH inhibitor URB597 both enhanced memory consolidation for inhibitory avoidance training. WIN55,212-22 effects on memory consolidation were predominantly mediated by CB1 receptor activation but CB2 receptors were involved as well. The URB597-induced memory enhancement was dependent on the activation not only of CB1 and CB2 receptors but, notwithstanding, PPAR-α and TRPV1 receptors were involved as well. Our findings drive beyond the classical hypothesis centered on the unique role of CB1 receptor activation for cannabinoid effects on memory, and reveal new insights in the neural mechanisms of memory consolidation.

## Introduction

It is known that cannabis users may experience euphoria, feelings of relaxation, altered perception of time, and increased appetite. Conversely, other users experience anxiety, fear, distrust, or panic (Mason and McBay, 1985; Thornicroft, 1990; Pope and Yurgelun-Todd, 1996; Huestis, 2002). Given the widespread distribution of cannabinoid receptors in many brain areas embodying the cortico-limbic system (e.g., prefrontal cortex, hippocampus, cerebellum, striatum, amygdala) (Katona et al., 2001; Mackie, 2005; Svizenska et al., 2008), it is not surprising that cannabinoids modulate emotional responses and emotional states, as well as cognitive and memory processes. Substantial evidence from both animal research and human studies showed that cannabinoids exert significant effects on attention and learning and memory, causing long-term modifications (Antonelli et al., 2005; Chhatwal et al., 2005; Quinn et al., 2008; Schweinsburg et al., 2008; Rubino et al., 2009; Campolongo et al., 2011; Batalla et al., 2013; Verrico et al., 2014). With regard to memory function, some reports showed disruptive effects (Riedel and Davies, 2005; Ranganathan and D’Souza, 2006; Solowij and Battisti, 2008), while others reported no evidence of cannabinoid-related deficits (Pope and Yurgelun-Todd, 1996; Solowij et al., 2002; Kanayama et al., 2004; Fisk and Montgomery, 2008). Contrasting findings on the behavioral effects induced by exogenous manipulation of the endocannabinoid signaling also arise from several preclinical studies (Morena and Campolongo, 2014). For instance, it has been shown that both cannabinoid agonists and antagonists exert in some studies anxiogenic-like effects, and anxiolytic-like effects in others regardless of their pharmacological action (Haller et al., 2004a,b; Moreira et al., 2006, 2009; Morena et al., 2016). Moreover, low versus high doses of cannabinoid agonists often induce opposite effects (e.g., anxiolytic versus anxiogenic effects) (Moreira and Wotjak, 2010), which were not reversed by cannabinoid receptor sub-type 1 (CB1) antagonists (Haller et al., 2007). Other *in vivo* studies demonstrated that administration of the non-selective cannabinoid receptor agonist WIN55,212-2 facilitates the consolidation of inhibitory avoidance memory, and the extinction of fear and spatial memory (Chhatwal et al., 2005; Pamplona et al., 2006; Campolongo et al., 2009b) while it impairs contextual fear conditioning acquisition (Pamplona and Takahashi, 2006), as well as consolidation and retrieval of spatial memory in rats (Yim et al., 2008; Morena et al., 2015). Administration of the endocannabinoid transporter inhibitor AM404 disrupted prepulse inhibition and enhanced the startle response but impaired memory recognition (Fernandez-Espejo and Galan-Rodriguez, 2004; Campolongo et al., 2012). On the other hand, increased anandamide signaling through inhibition of its metabolizing enzyme FAAH by URB597 treatment, enhanced consolidation and reconsolidation of aversive memories (Morena et al., 2014; Ratano et al., 2014).

One possible explanation for such contrasting results could lie on the fact that cannabinoid receptors are expressed at both glutamatergic and GABAergic synapses, which often exert opposite effects on cognition and emotions (Ruehle et al., 2012). Discrepant findings could also be due to differences in the expression, distribution and functional characteristics of cannabinoid receptors (Callen et al., 2012), as well as to activation of distinct neuronal circuits depending upon the complexity of the behavioral tasks used. Moreover, the effects induced by pharmacological manipulation of endocannabinoid neurotransmission are strongly influenced by environmental and experimental conditions (Zanettini et al., 2011; Campolongo et al., 2012; Manduca et al., 2014; Morena and Campolongo, 2014). Similarly, the time of drug administration should be considered as a further confounding factor, as pre- versus post-training, and pre- versus post-retrieval administration may influence distinctive memory functions (i.e., memory acquisition, consolidation, retrieval or extinction) (Morena and Campolongo, 2014). In particular, pre-training administration may affect several other parameters such as pain sensitivity and motivation, among several others, rather than memory functions *per se* leading to a wrong interpretation of the obtained results.

Until now the cognitive effects induced by cannabinoids drugs have been considered to be dependent only on CB1 activation, overlooking a possible involvement of CB2 receptors. Traditionally, CB2 receptors have been thought to be exclusively expressed in the cells of the immune system (Munro et al., 1993). Despite their expression in neurons is still controversial, growing evidence strongly suggests that CB2 receptors are also expressed in the brain, and that they are involved in several neurobiological functions (Gong et al., 2006; Onaivi et al., 2006; Brusco et al., 2008; Garcia-Gutierrez et al., 2012; Navarrete et al., 2012; Li and Kim, 2016). Noteworthy, endocannabinoids, and their analogs, show binding affinity for other receptor families beyond the cannabinoid receptors, including the peroxisome proliferator-activated receptors (PPARs) (Fu et al., 2003; Bouaboula et al., 2005; O’Sullivan, 2007; Campolongo et al., 2009a; Luchicchi et al., 2010), and the transient receptor potential channels, especially vanilloid receptors transient receptor potential cation channel subfamily V member 1 (TRPV1) (Zygmunt et al., 1999; Di Marzo and De Petrocellis, 2010). PPARs are family of a nuclear hormone receptor (PPAR-α, PPAR-β/δ, and PPAR-γ) which regulate several biological functions such as lipid homeostasis (Friedland et al., 2012; Menendez-Gutierrez et al., 2012; Neher et al., 2012; Poulsen et al., 2012; Alexander et al., 2015). Particularly, PPAR-α is expressed in the hippocampus and regulates the expression of neuronal cAMP-response-element binding protein (CREB), a key regulator of memory formation (Roy et al., 2013, 2015). Consistently, PPAR-α knockout mice showed an impairment in hippocampal-dependent memory and in spatial learning.

Transient receptor potential cation channel subfamily V member 1 is a calcium-permeable cation channel known to be involved in regulating both long-term potentiation (LTP) and long-term depression (LTD) in the hippocampus (Marsch et al., 2007; Li et al., 2008; Chavez et al., 2010; Bennion et al., 2011). Moreover, mice lacking TRPV1 receptors showed reduced freezing response in the auditory fear conditioning as well as reduced anxiety-like behaviors compared to wild-type (Marsch et al., 2007). On the other hand, activation of TRPV1 locally into the hippocampus counteract the deleterious effects of stress on spatial memory retrieval (Li et al., 2008).

Therefore, the aim of the present study was to investigate the effects induced by cannabinoid compounds with different target selectivity on memory consolidation for aversive experiences, and to determine whether such effects are solely mediated by CB1 receptors, or if other non-CB1 targets might also be involved. Rats were trained in an inhibitory avoidance task, and the impact of any possible confounding variable has been reduced to the minimum by using the identical behavioral setting for any tested drug (e.g., animals were all tested in the same behavioral equipment at the same time of the day and all equally handled, drugs were all dissolved in an identical vehicle). To selectively test the effects on memory consolidation, all drugs were systemically administered post-training. Thereafter, we evaluated the involvement of different receptors, such as the PPAR-α, TRPV1 or CB2 receptors, and not only the classic CB1 activation in mediating the effects of the tested drugs on memory.

## Experimental Procedures

### Animals

Male adult Sprague-Dawley rats (total *n* = 499; 350–450 g at the time of training; Charles River Laboratories, Calco, Italy) were housed individually and maintained in a temperature-controlled environment (20 ± 1°C) under a 12-h light/12-h dark cycle (7:00 AM to 7:00 PM lights on) with unlimited access to food and water. All procedures involving animal care or treatments were approved by the Italian Ministry of Health (Rome, Italy) and performed in compliance with the guidelines of the Directive 2010/63/EU of the European Parliament, and the D. L. 26/2014 of Italian Ministry of Health.

### Drug Treatments

The cannabinoid receptor agonist WIN55,212-2 [R(+)-[2,3-dihydro-5-methyl-3-[(morpholinyl) methyl] pyrolol [1,2,3-de]-1,4-benzoxazin-yl]-(1-naphthalenyl) methanone mesylate] (0.3, 1, and 3 mg/kg); the FAAH inhibitor URB597 [(3′-(aminocarbonyl)[1,1′-biphenyl]-3-yl)-cyclohexylcarbamate] (0.1, 0.2, and 0.3 mg/kg); the CB1 receptor antagonist SR141716 [5-(4-chloro-phenyl)-1-(2,4-dichlorophenyl)-4-methyl-N-1-piperidinyl-1H-pyrazole-3-carboxamide] (0.3, 1, and 3 mg/kg); the CB2 receptor antagonist SR144528 [5-(4-chloro-3-methylphenyl)-1-[(4-methylphenyl)methyl]-N-[(1S,2S,4R)-1,3,3-trimethylbicyclo[2.2.1]hept-2-yl]-1H-pyrazole-3-carboxamide] (0.03, 0.1, and 0.3 mg/kg); the PPAR-α receptor antagonist GW6471 [N-[(2S)-2-[[(1Z)-1-methyl-3-oxo-3-[4-(trifluoromethyl)phenyl]-1-propen-1-yl]amino]-3-[4-[2-(5-methyl-2-phenyl-4-oxazolyl)ethoxy]phenyl]propyl]propanamide] (1, 2, and 4 mg/kg, Tocris Bioscience), capsazepine (5 mg/kg, Tocris Bioscience) were administered by intraperitoneal injection in a volume of 1 ml/kg immediately *after* the training trial. Drug solutions, freshly prepared before each experiment, were dissolved all in the same vehicle containing 5% polyethylene glycol, 5% Tween-80 and 90% saline. WIN55,212-2, URB597, SR141716, SR144528 were granted by the NIMH Chemical Synthesis and Drug Supply Program. Time for drug administration has been chosen on the basis of our preliminary findings or literature data taking into account the pharmacokinetic properties of all drugs (Lichtman et al., 1995; Bridges et al., 2001; Capasso et al., 2001; Da and Takahashi, 2002; Pamplona and Takahashi, 2006; Morgese et al., 2007; Russo et al., 2007; Butler et al., 2008; Sagar et al., 2008; Campolongo et al., 2009a; Polissidis et al., 2009; Costa et al., 2010; Adamczyk et al., 2012; Scuderi et al., 2014).

### Inhibitory Avoidance Apparatus and Procedure

Rats were trained and tested in an inhibitory avoidance apparatus consisting of two compartments, separated by a sliding door. The starting compartment (31 cm long), made of opaque white plastic, was illuminated by a lamp; the shock compartment (60 cm long), made of two dark, electrifiable metal plates, was not illuminated (McGaugh et al., 1988). Training and testing were performed during the light phase, between 10:00 AM and 2:00 PM, and were conducted in dim light conditions in a sound-attenuated room. Animals were handled 1 min each for 3 days prior to the training day.

The behavioral procedure was performed as previously described (Morena et al., 2014). Briefly, for training, the rats were placed into the starting compartment of the apparatus, facing away from the door, and were permitted to explore the apparatus. After the rats stepped completely into the dark compartment, the sliding door was closed and a single inescapable footshock was delivered (0.35 mA, 1 s). Fifteen seconds after termination of the footshock the animals were removed from the shock compartment. Retention was tested 48 h later. On the retention test trial, the rats were placed into the starting compartment and the latency to reenter the shock compartment was recorded (cut-off 600 s) and used as a measure of memory retention. Longer latencies were interpreted as indicating better memory retention (Dawson and McGaugh, 1971). Between each session the apparatus was cleaned with a 70% ethanol solution.

### Statistical Analysis

Data were analyzed using ANOVA with treatment as the between-subject factor using standard statistical software (SPSS 23.0). To determine whether learning had occurred, paired *t*-tests were used to compare the training and retention latencies of the vehicle groups. The source of the detected significances was determined by Tukey–Kramer’s *post hoc* tests. *P*s < 0.05 were considered statistically significant. The number of rats per group is indicated in the figures. Data are expressed as mean ± SEM.

## Results

### Effect of Post-training Administration of WIN55,212-2 on Inhibitory Avoidance Retention Performances

In this experiment we examined whether the non-selective cannabinoid receptor agonist WIN55,212-2, administered immediately after the training of the inhibitory avoidance task, would affect retention performance at testing. Average step-through latencies for all groups during the training, before footshock and drug administration, were 13.70 ± 1.25 s. One-way ANOVA for training latencies revealed no significant differences among groups (*F*_3,42_ = 1.45, *p* = 0.24). At testing, retention latencies of rats given vehicle immediately after the training were significantly longer than their approach latencies during the training trial (*t* = 2.53, *p* = 0.03), showing that vehicle treated rats correctly retained the memory of the footshock received during the training.

**FIGURE 1:**
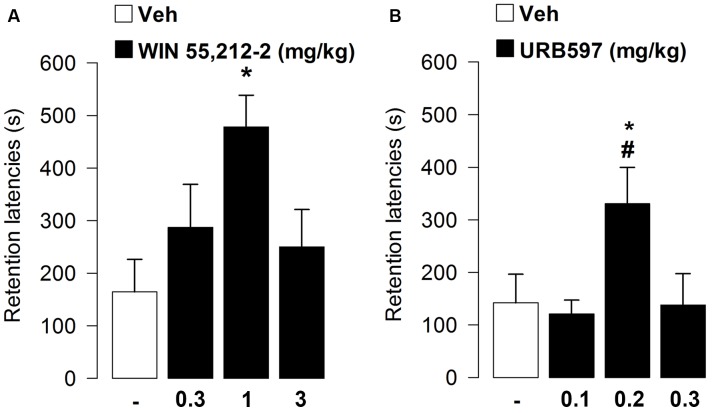
**Effects of post-training administration of WIN55,212-2 or URB597 on retention latencies in the inhibitory avoidance task**. WIN55,212-2 1 mg/kg **(A)** and URB597 0.2 mg/kg **(B)** increased retention latencies showing a facilitation of memory retention. Data represent mean ± SEM. ^∗^*p* < 0.05 vs. vehicle-treated rats; ^#^*p* < 0.05 vs. URB597-treated rats (*n* = 11–13 per group).

Retention latencies analyzed by one-way ANOVA revealed a significant treatment effect (*F*_3,42_ = 3.44, *p* = 0.03). *Post hoc* analysis indicated that rats administered with WIN55,212-2 at the dose of 1 mg/kg had retention latencies significantly longer than vehicle-treated rats (*p* < 0.05; **Figure 1A**), thus showing that the non-selective cannabinoid receptor agonist WIN55,212-2 enhanced memory consolidation of inhibitory avoidance training.

### Effect of Post-training Administration of URB597 on Inhibitory Avoidance Retention Performances

In this experiment we examined whether enhancing anandamide signaling at active synapses, by administering the FAAH enzyme inhibitor URB597 immediately after the training of the inhibitory avoidance task, would affect retention performance at testing. Average step-through latencies for all groups during the training, before footshock and drug administration, were 12.91 ± 1.24 s. One-way ANOVA for training latencies revealed no significant differences among groups (*F*_3,44_ = 1.25, *p* = 0.30).

At testing, retention latencies of rats given vehicle were significantly longer than their approach latencies during the training trial (*t* = -2.45, *p* = 0.03), showing that the rats retained the memory of the footshock. Retention latencies analyzed by one-way ANOVA revealed a significant treatment effect (*F*_3,44_ = 4.01, *p* = 0.01). *Post hoc* analysis indicated that rats administered with URB597, at the dose of 0.2 mg/kg, had retention latencies significantly longer than vehicle-treated rats (*p* < 0.05; **Figure 1B**), thus showing that enhancing anandamide tone at active synapses enhanced memory consolidation of aversive training.

### Effect of Post-training Administration of Cannabinoid Receptor Antagonists on Inhibitory Avoidance Retention Performances

In a first experiment we examined whether blocking CB1 receptor signaling by administering the CB1 receptor antagonist SR141716 immediately after the training of the inhibitory avoidance task would affect retention performance at testing. Average step-through latencies for all groups during the training, before footshock and drug administration, were 12.60 ± 1.49 s. One-way ANOVA for training latencies revealed no significant differences among groups (*F*_3,39_ = 1.62, *p* = 0.20).

At testing, retention latencies of rats given vehicle immediately after the training were significantly longer than their approach latencies during the training trial (*t* = -4.25, *p* < 0.001), showing that the rats retained the memory of the footshock.

**FIGURE 2:**
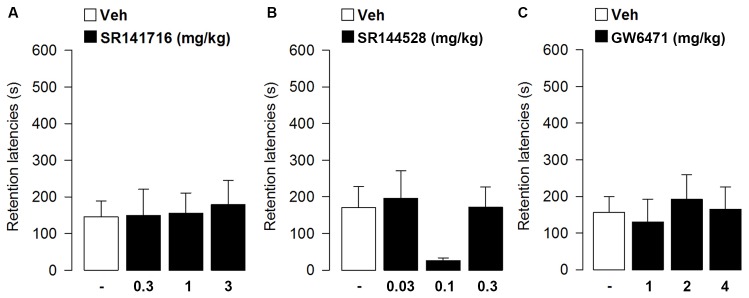
**Effects of post-training administration of CB1 or CB2 or PPAR-α antagonists on retention latencies in the inhibitory avoidance task**. SR141716 **(A)**, SR144528 **(B)** and GW6471 **(C)** did not affect retention latencies in the inhibitory avoidance task. Data represent mean ± SEM (*n* = 10–13 per group).

Retention latencies analyzed by one-way ANOVA did not reveal a significant treatment effect (*F*_3,39_ = 0.06, *p* = 0.98; **Figure 2A**), thus showing that blocking CB1 receptor signaling did not affect memory consolidation for aversive events.

In a second experiment we examined whether blocking CB2 receptor signaling by administering the CB2 receptor antagonist SR144528 immediately after the training of the inhibitory avoidance task would affect retention performance at testing. Average step-through latencies for all groups during the training, before footshock and drug administration, were 15.05 ± 1.59 s. One-way ANOVA for training latencies revealed no significant differences among groups (*F*_3,43_ = 1.59, *p* = 0.21; **Figure 2B**). At testing, retention latencies of rats given vehicle immediately after the training were significantly longer than their approach latencies during the training trial (*t* = -2.62, *p* = 0.03), showing that the rats retained the memory of the footshock received during the training. Retention latencies analyzed by one-way ANOVA did not reveal a significant treatment effect (*F*_3,40_ = 1.62, *p* = 0.20), thus showing that blocking CB2 receptor signaling did not affect memory consolidation for aversive events. However, it should be noted that although it is not statistically significant, SR144528 at the dose of 0.1 mg/kg, tends to impair memory retention (**Figure 2B**).

### Effect of Post-training Administration of GW6471 on Inhibitory Avoidance Retention Performances

In this experiment we examined whether blocking PPAR-α receptor signaling by administering the PPAR-α antagonist GW6471 immediately after the training of the inhibitory avoidance task would affect retention performance at testing. Average step-through latencies for all groups during the training, before footshock and drug administration, were 14.54 ± 1.43 s. One-way ANOVA for training latencies revealed no significant differences among groups (*F*_3,38_ = 1.37, *p* = 0.27; **Figure 2C**). At testing, retention latencies of rats given vehicle immediately after the training were significantly longer than their approach latencies during the training trial (*t* = -3.37, *p* = 0.01), showing that the rats retained the memory of the footshock received during the training. Retention latencies analyzed by one-way ANOVA did not reveal a significant treatment effect (*F*_3,38_ = 0.19, *p* = 0.90), thus showing that blocking PPAR-α receptor signaling did not affect memory consolidation for aversive events.

### Enhancement of Inhibitory Avoidance Retention Performances Induced by WIN55,212-2 Requires Concomitant Activation of both CB1 and CB2 Receptors

In these experiments we examined whether the enhancing effect on memory consolidation induced by WIN55,212-2 could depend on activation of CB1 or CB2 receptors. First, we co-administered immediately after the training of the inhibitory avoidance the memory-modulating dose of WIN55,212-2 (1 mg/kg) with SR141716 (0.3 mg/kg). Average step-through latencies for all groups during the training, before footshock and drug administration, were 13.27 ± 1.37 s. One-way ANOVA for training latencies revealed no significant differences among groups (*F*_3,34_ = 0.58, *p* = 0.63).

**FIGURE 3:**
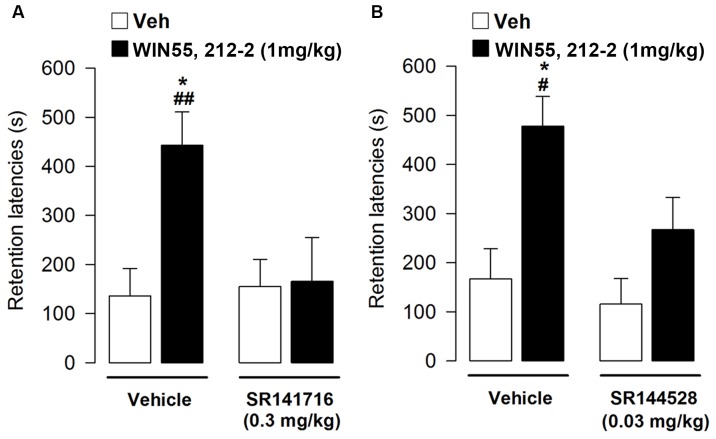
**Effects of post-training administration of WIN55,212-2 after concurrent administration with CB1 or CB2 antagonists on retention latencies in the inhibitory avoidance task**. The enhancing effect on retention latencies induced by WIN55,212-2 1 mg/kg was reverted by co-administration of WIN55,212-2 1 mg/kg with: **(A)** SR141716 (0.3 mg/kg); **(B)** SR144528 (0.03 mg/kg). Data represent mean ± SEM. ^∗^*p* < 0.05 vs. vehicle-treated rats; ^#^*p* < 0.05 vs. WIN55,212-2+SR144528; ^##^*p* < 0.01 vs. WIN55,212-2+SR141716 (*n* = 9–12 per group).

At testing, retention latencies of vehicle-treated rats were significantly longer than their approach latencies during the training trial (*t* = -2.24, *p* = 0.49), showing that the rats retained the memory of the footshock received during the training. Interestingly, retention latencies analyzed by two-way ANOVA revealed a significant agonist effect (*F*_1,34_ = 5.72, *p* = 0.02), a non-significant antagonist effect (Vehicle or SR141716) (*F*_1,34_ = 3.81, *p* = 0.06) and a significant treatment interaction (*F*_1,34_ = 4.99, *p* = 0.03). *Post hoc* analysis indicated that rats administered with WIN55,212-2 had retention latencies significantly longer when compared with vehicle-treated rats (*p* < 0.01; **Figure 3A**) or with rats co-administered with WIN55,212-2 and SR141716. Thus, the enhancing effect on memory consolidation induced by WIN55,212-2 is mediated by CB1 receptor activation.

In a second set of experiment we co-administered immediately after the training of the inhibitory avoidance the memory-modulating dose of WIN55,212-2 (1 mg/kg) with SR144528 (0.03 mg/kg). Average step-through latencies for all groups during the training, before footshock and drug administration, were 13.71 ± 1.10 s. One-way ANOVA for training latencies revealed no significant differences among groups (*F*_3,41_ = 1.63, *p* = 0.02). At testing, retention latencies of rats given vehicle immediately after the training were significantly longer than their approach latencies during the training trial (*t* = -2.55, *p* = 0.2), showing that the rats acquired the task. Interestingly, retention latencies analyzed by two-way ANOVA revealed a significant agonist or antagonist effect (*F*_1,41_ = 14.63, *p* = 0.0004; *F*_1,41_ = 4.70, *p* = 0.04, respectively), but not a significant treatment interaction (*F*_1,41_ = 1.75, *p* = 0.19). *Post hoc* analysis showed that rats co-treated with vehicle and WIN55,212-2 had longer retention latencies that rats given vehicle alone or vehicle and SR144528 together (*p* < 0.05; **Figure 3B**). Thus, the enhancing effect on memory consolidation induced by WIN55,212-2 depends not only CB1 receptor activation but requires CB2 receptor activation as well.

### Enhancement of Inhibitory Avoidance Retention Performances Induced by URB597 Requires Concomitant Activation of both CB1 and CB2 Receptors

In these experiments we examined whether the enhancing effect on retention latencies induced by URB597 could depend on activation of cannabinoid receptors. In a first set of experiment we co-administered immediately after the training of the inhibitory avoidance the memory-modulating dose of URB597 (0.2 mg/kg) with SR141716 (0.3 mg/kg). Average step-through latencies for all groups during the training, before footshock and drug administration, were 12.43 ± 1.92 s. One-way ANOVA for training latencies revealed no significant differences among groups (*F*_3,41_ = 0.56, *p* = 0.64).

**FIGURE 4:**
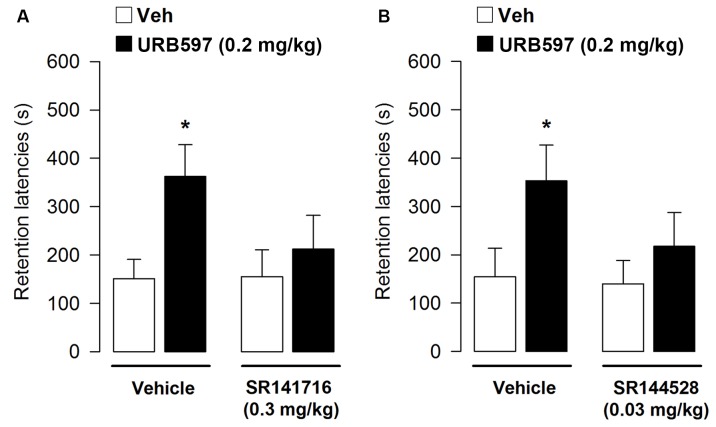
**Effects of post-training administration of URB597 after concurrent administration with the CB1 and CB2 antagonists on retention latencies in the inhibitory avoidance task**. The enhancing effect on memory retention induced by URB597 0.2 mg/kg was reverted by blocking cannabinoid receptor activity through co-administration with: **(A)** SR141716 (0.3 mg/kg); **(B)** SR144528 (0.03 mg/kg). Data represent mean ± SEM. ^∗^*p* < 0.05 vs. vehicle-treated rats (*n* = 10–12 per group).

At testing, retention latencies of vehicle-treated were significantly longer than their approach latencies during the training trial (*t* = –3.54, *p* = 0.005), showing that the rats retained the memory of the footshock received during the training. Interestingly, retention latencies analyzed by two-way ANOVA revealed a significant effect of URB597 (*F*_1,41_ = 5.21, *p* = 0.03), a non-significant SR141716 effect (*F*_1,41_ = 1.55, *p* = 0.22) and a non-significant treatment interaction (*F*_1,41_ = 1.72, *p* = 0.22). *Post hoc* analysis indicated that rats administered with vehicle and URB597 had retention latencies significantly longer when compared with vehicle-treated rats (*p* < 0.05; **Figure 4A**) but they did not significantly differ from rats co-administered with URB597 and SR141716. However, retention latencies of rats treated with URB597 and SR141716 were comparable to the latencies of controls. Thus, the enhancing effect on memory consolidation induced by URB597 is only partially dependent on CB1 receptor activation. In a second set of experiment we co-administered immediately after the training of the inhibitory avoidance the memory-modulating dose of URB597 (0.2 mg/kg) with a dose not altering memory *per se* of SR144528 (0.03 mg/kg). Average step-through latencies for all groups during the training, before footshock and drug administration, were 11.60 ± 1.24 s. One-way ANOVA for training latencies revealed no significant differences among groups (*F*_3,38_ = 2.54, *p* = 0.07).

At testing, retention latencies of rats given vehicle immediately after the training were significantly longer than their approach latencies during the training trial (*t* = -6.14, *p* < 0.0001), showing that the rats acquired the task. Retention latencies analyzed by two-way ANOVA revealed a significant URB597 effect (*F*_1,38_ = 4.80, *p* = 0.03), but not a significant SR144528 or interaction effect (*F*_1,38_ = 1.41, *p* = 0.24; *F*_1,38_ = 0.93, *p* = 0.34, respectively). *Post hoc* analysis indicated that rats administered with vehicle and URB597 had retention latencies significantly longer when compared with vehicle-treated rats (*p* < 0.05; **Figure 4B**) even though they did not significantly differ from rats co-administered with URB597 and SR144528. However, the retention latencies displayed by rats treated with URB597 and SR144528 were comparable to the latencies of control animals. Thus, the enhancing effect on memory consolidation induced by URB597 is also partially dependent on CB2 receptor activation.

### Enhancement of Inhibitory Avoidance Retention Performances Induced by URB597 Requires PPAR-α Receptor Activation

**FIGURE 5:**
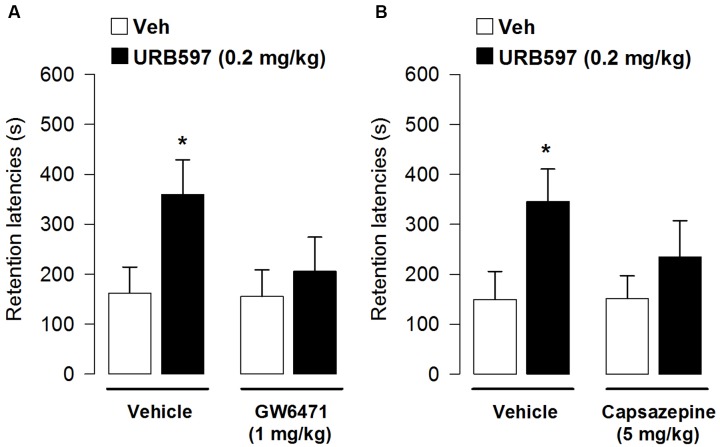
**Effects of post-training administration of URB597 after concurrent administration with PPAR-α or TRPV1 antagonists on retention latencies**. The enhancing effect on retention latencies induced by URB597 0.2 mg/kg was partially reverted by blocking PPAR-α **(A)** or TRPV1 **(B)**. Data represent mean ± SEM. ^∗^*p* < 0.05 vs. vehicle-treated rats (*n* = 10–12 per group).

In this experiment we examined whether the enhancing effect on retention latencies induced by URB597 could depend on activation of PPAR-α receptors. We co-administered immediately after the training of the memory-modulating dose of URB597 (0.2 mg/kg) with a dose not altering memory *per se* of GW6471 (1 mg/kg). Average step-through latencies for all groups during the training, before footshock and drug administration, were 12.20 ± 1.20 s. One-way ANOVA for training latencies revealed no significant differences among groups (*F*_3,43_ = 1.52, *p* = 0.22). At testing, retention latencies of rats given vehicle were significantly longer than their approach latencies during the training trial (*t* = -2.94, *p* = 0.01), showing that the rats retained the memory of the footshock received during the training. Retention latencies analyzed by two-way ANOVA revealed a significant URB597 effect (*F*_1,43_ = 4.13, *p* = 0.04), but not a significant SR141716 or interaction effect (*F*_1,43_ = 1.72, *p* = 0.20; *F*_1,43_ = 1.45, *p* = 0.24, respectively). *Post hoc* analysis indicated that rats administered with vehicle and URB597 had retention latencies significantly longer when compared with vehicle rats (*p* < 0.05; **Figure 5A**) but they did not significantly differ from rats co-administered with URB597 and GW6471. However, at the same time the difference between rats given vehicles and rats given URB597 and GW6471 is not statistically significant. Thus, the enhancing effect on memory consolidation induced by URB597 could be partially dependent on PPAR-α receptor activation.

### Enhancement of Inhibitory Avoidance Retention Performances Induced by URB597 Requires Activation of TRPV1 Receptors

In this experiment we examined whether the enhancing effect on retention latencies induced by URB597 could depend on activation of TRPV1 receptors. We co-administered immediately after the training of the memory-modulating dose of URB597 (0.2 mg/kg) with a dose not altering memory *per se* of capsazepine (5 mg/kg). Average step-through latencies for all groups during the training, before footshock and drug administration, were 15.67 ± 1.39 s. One-way ANOVA for training latencies revealed no significant differences among groups (*F*_3,42_ = 1.27, *p* = 0.30).

At testing, retention latencies of vehicle-treated rats were significantly longer than their approach latencies during the training trial (*t* = -6.48, *p* < 0.0001), showing that the rats retained the memory of the footshock received during the training. Retention latencies analyzed by two-way ANOVA revealed a significant URB597 effect (*F*_1,42_ = 5.55, *p* = 0.02), a non-significant capsazepine effect (*F*_1,42_ = 0.84, *p* = 0.36) and a non-significant treatment interaction (*F*_1,43_ = 0.91, *p* = 0.35). *Post hoc* analysis indicated that rats administered with vehicle and URB597 had retention latencies significantly longer when compared with vehicle-treated rats (*p* < 0.05; **Figure 5B**) but they did not significantly differ from rats co-administered with URB597 and capsazepine. However, no statistically significant difference was found between rats given vehicle and rats given URB597 and capsazepine. Therefore, we can hypothesize that the enhancing effect on memory consolidation induced by URB597 could also partially require TRPV1 receptor activation.

## Discussion

The present findings show that direct activation of cannabinoid receptors, or pharmacological-induced potentiation of the endocannabinoid tone, both enhance memory consolidation for aversive experiences. We provide the first demonstration that these effects are not only mediated by the activation of CB1 receptors, but that CB2 receptors are also involved. We further show that TRPV1 and PPAR-α receptors are involved in mediating endocannabinoid effects on memory.

The memory facilitation effect induced by WIN55,212-2 and the FAAH inhibitor URB597 is in line with previous studies showing that WIN55,212-2 or URB597 induce in rats, through a CB1-dependent signaling, enhancing effects when locally infused into the basolateral complex of the amygdala (BLA), into the hippocampus and into the prefrontal cortex immediately after the training of an inhibitory avoidance task (Campolongo et al., 2009b; Morena et al., 2014). However, opposite effects have been reported after systemic or central administration of cannabinoid agonists. For instance, it has been shown that systemic administration of WIN55,212-2 or URB597 attenuated memory consolidation in rats and mice exposed to different cognitive tasks (Mackowiak et al., 2009; Busquets-Garcia et al., 2011; Galanopoulos et al., 2014; Hasanein and Teimuri Far, 2015; Kruk-Slomka et al., 2016a). Similarly, post-training central activation of cannabinoid receptors induced an amnesic response in rats exposed to the Inhibitory Avoidance, contextual fear conditioning, Morris Water Maze or object recognition tasks (Clarke et al., 2008; Moshfegh et al., 2011; Segev and Akirav, 2011; Hasanein and Sharifi, 2015). However, other reports demonstrate that administration of the CB1 antagonist AM251 immediately after training, induced similar effects to those induced by agonists on the consolidation of memory in the inhibitory avoidance or contextual fear conditioning tasks (Bucherelli et al., 2006; Campolongo et al., 2009b). Several confounding variables could be responsible for these apparent discrepancies. Among them, the different experimental context/conditions, the drug selectivity, the vehicle used for drug dissolution and the time of administration are of crucial importance (Haller et al., 2004a; Varga et al., 2008; Campolongo et al., 2012, 2013; Kruk-Slomka et al., 2016b). For instance, following a pre-training administration, cannabinoid compounds could strongly interfere with pain perception (Burston and Woodhams, 2014) and/or locomotor activity at the time of training (Martin and Lichtman, 1998). Moreover, each type of behavioral task could activate distinctive neural substrates. Together with the extensive and heterogeneous pattern of cannabinoid receptor expression at brain level all these elements might explain the differences in the effect induced by systemic or local infusion of cannabinoid drugs.

Up-to-date the cognitive effects of synthetic or endogenous cannabinoids have been considered as mostly mediated by CB1 receptors expressed in the nervous system and CB2 receptors expressed in the immune system (Devane et al., 1988; Matsuda et al., 1990; Munro et al., 1993). However, recent evidence indicates that CB2 receptors are also expressed in the brain. CB2 receptors (proteins or mRNA) have been found in various areas of the central nervous system, such as the brainstem, pons, cerebellum, cerebral cortex, hippocampus, amygdala, striatum, substantia nigra, thalamus, hypothalamus and olfactory bulb (Svizenska et al., 2008; Atwood and Mackie, 2010). Immunostaining studies demonstrated that CB2 receptors are widely expressed in the soma and dendrites of pyramidal cells and in some interneurons in the hippocampus (Gong et al., 2006; Onaivi et al., 2006; Brusco et al., 2008), as well as in microglia (Svizenska et al., 2008; Atwood and Mackie, 2010). In the dendrites of hippocampal neurons, CB2 receptors locate near synaptic contacts (Solowij et al., 2002; Onaivi et al., 2006; Brusco et al., 2008). Despite the role of CB1 receptors in the regulation of neurophysiological functions has been extensively characterized, the presence of CB2 receptors in the brain is a novel finding, and their functional significance remain to be clarified. Electrophysiological and morphological studies strongly suggest that CB2 receptors are involved in synaptic transmission and plasticity (Li and Kim, 2016). Consequently, it has been demonstrated that CB2 receptors also modulate neurophysiological functions and behaviors such as anxiety (Garcia-Gutierrez et al., 2012), impulsive behaviors (Navarrete et al., 2012), vomiting (Van Sickle et al., 2005), and pain (Jhaveri et al., 2007; Anand et al., 2009; Han et al., 2013). It has been also demonstrated that CB2 receptor knockout, knock-down or overexpression induce phenotypes resembling those seen in several neuropsychiatric disorders (Onaivi et al., 2008; Racz et al., 2008; Busquets-Garcia et al., 2011; Ortega-Alvaro et al., 2011; Aracil-Fernandez et al., 2012; Romero-Zerbo et al., 2012; Garcia-Gutierrez et al., 2013), while blocking endocannabinoid degradation reduces anxiety via CB2 receptor activation (Busquets-Garcia et al., 2011).

Brain CB1 and CB2 receptors can be both activated by cannabinoids. Anandamide and 2-AG, both substrates for FAAH enzymes (Bisogno et al., 2002), act as full agonist and partial agonist, respectively (Mackie and Hille, 1992; Mechoulam et al., 1995; Showalter et al., 1996; Sugiura et al., 2000), with a 3- to 4-fold higher affinity for the CB1 than for the CB2 receptor (Mechoulam et al., 1995; Showalter et al., 1996). Δ^9^-THC, the main psychoactive constituent of *Cannabis sativa*, binds to CB1 and CB2 receptors with the same affinity (Showalter et al., 1996). So that, it is tentative to hypothesize that when the levels of endogenous cannabinoids are elevated, or after marijuana consumption, both CB1 and CB2 receptors could be differentially recruited to contribute to the effects of (endo)cannabinoid on memory. Based on these premises, in the present work, we systemically administered different cannabinoid compounds immediately after inhibitory avoidance training. We found that immediate post-training administration of URB597 induced facilitates memory retention. Not only the CB1 antagonist SR141716 but, interestingly, also the CB2 antagonist SR144528 abolished this effect, thus demonstrating that the URB597-induced facilitation of memory consolidation requires a concurrent activation of both CB1 and CB2 receptors.

It is known that several cannabinoid compounds could activate not only CB1 and CB2 receptors but also PPARα (Fu et al., 2003; Bouaboula et al., 2005; O’Sullivan, 2007; Campolongo et al., 2009a; Luchicchi et al., 2010) and TRPV1 receptors (Zygmunt et al., 1999; Svizenska et al., 2008; Di Marzo and De Petrocellis, 2010). Here we show that URB597 exerts its effect on memory consolidation by additionally activating PPAR-α and TRPV1 as well. It should be taken into account that FAAH, besides anandamide, hydrolyses other N-acylethanolamines, such as palmitoylethanolamide (PEA) and oleoylethanolamide (OEA) (Bisogno et al., 2002), which in turn activate PPARs receptors. PPARs are a nuclear hormone receptor family of and their target genes are involved in the maintenance of both metabolism and energy homeostasis, inflammation, and cell differentiation (Friedland et al., 2012; Menendez-Gutierrez et al., 2012; Neher et al., 2012; Poulsen et al., 2012; Alexander et al., 2015). In the last decade, growing evidence showed that PPARs are bound and activated by endocannabinoids, endocannabinoid-like compounds, phytocannabinoids and synthetic cannabinoid postulating a potential roles for PPAR activation in the physiological effects of cannabinoids (Liu et al., 2003; O’Sullivan, 2007; O’Sullivan et al., 2012). Although there is limited evidence concerning the role of PPAR-α on memory and cognition, our results are in line with previous studies showing that systemic administrations of OEA, URB597, or WY14643 (a PPAR-α agonist) facilitate learning processes and enhance retention of inhibitory avoidance task through activation of PPAR-α (Campolongo et al., 2009a; Mazzola et al., 2009). Luchicchi et al. (2010) reported that URB597 specifically modulates neuronal responses to different substances of abuse through actions on both PPAR-α receptors and cannabinoid CB1 receptors, suggesting that the fatty acid ethanolamides anandamide, OEA, and PEA are engaged in the modulation of neurophysiological and behavioral effects, at least for addictive drugs. Several models on the potential mechanisms of cannabinoid/PPAR interactions have been proposed: (1) cannabinoids could bind directly to PPARs and be converted into PPAR-active metabolites; (2) activation of cell surface cannabinoid receptors could trigger intracellular signaling cascades that lead to an indirect activation of PPARs; (3) cannabinoids may be actively transported to the nucleus by intracellular lipid binding proteins, the Fatty Acid Binding Proteins (FABPs), to interact with PPAR-α (Hughes et al., 2015; O’Sullivan, 2016). Further investigations are needed in order to clarify which of the mechanisms described above might be involved in the potentiation of memory consolidation driven by cannabinoid/PPAR interaction.

The present results further show that not only CB and PPARs but also TRPV1 receptors are involved in the potentiation of memory consolidation for aversive experiences. Anandamide is a full agonist of TRPV1 (Zygmunt et al., 1999), and emerging evidence strongly suggests that anandamide signaling modulates synaptic plasticity via post-synaptic TRPV1 activation (Chavez et al., 2010; Grueter et al., 2010; Puente et al., 2011; Chavez et al., 2014). Furthermore, anandamide inhibits phasic endocannabinoid signaling via TRPV1 activation and 2-AG synthesis mediated by metabotropic glutamate receptor activation (Maccarrone et al., 2008). It is known that TRPV1 are implicated in LTD and LTP in the hippocampus (Marsch et al., 2007; Chavez et al., 2010; Bennion et al., 2011), and that they modulate hippocampal-dependent memory in rats trained in the fear conditioning and step-down inhibitory avoidance tasks (Genro et al., 2012). Moreover, it has been reported that TRPV1 receptors may participate in the modulation of emotional states, as TRPV1-deficient mice when tested in the elevated plus maze and dark-light box tasks showed less anxiety-like behavior than wild-type littermate (Marsch et al., 2007). *In vitro* and *in vivo* studies reported that the TRPV1 agonist capsaicin both facilitated LTP and prevented spatial memory retrieval deficit, while the selective antagonist capsazepine inhibited LTD (Li et al., 2008). Moreover, Batista et al. (2015) reported that anandamide produced anxiolytic-like effect via TRPV1 receptor activation. Here we add to these observations the finding that TRPV1 receptors participate in the modulation of memory consolidation for emotional experiences exerted by cannabinoid compounds.

Taken together, the results of the present study show that the facilitation of memory consolidation induced by increased levels of endogenous cannabinoids is dependent not only upon cannabinoid receptor activation but involves multiple neurotransmission pathways. This evidence drives beyond the classical hypothesis centered on the unique role of CB1 receptors on memory modulation by cannabinoids, and reveals new insights in the neural mechanisms of emotional memory.

## Author Contributions

All authors contributed to and have approved the final manuscript. PR contributed to the design of the experiments, performed the experiments, and wrote the manuscript. PC supervised the project, designed the experiments and wrote the manuscript. VT and MP contributed to the design of the experiments and revised the manuscript. All authors read and approved the final version of the manuscript.

## Conflict of Interest Statement

The authors declare that the research was conducted in the absence of any commercial or financial relationships that could be construed as a potential conflict of interest.
